# Keloid Development After Fine Needle Aspiration of the Thyroid: A Rare Case and Review of Management Strategies

**DOI:** 10.7759/cureus.42359

**Published:** 2023-07-24

**Authors:** Shaniah S Holder, Alaerebo S Malvan-iyalla, Sara Arfan, Vimal Basani, Frederick Tiesenga

**Affiliations:** 1 Medicine, American University of Barbados School of Medicine, Bridgetown, BRB; 2 General Surgery, Windsor University School of Medicine, Chicago, USA; 3 Medicine, St. George's University School of Medicine, True Blue, GRD; 4 General Surgery, West Suburban Medical Center, Chicago, USA

**Keywords:** medical and cosmetic dermatology, fine-needle aspiration, african american/black, pathological scar, keloid

## Abstract

Keloids are pathological scars characterized by abnormal proliferation of tissue as a result of cutaneous injury. There is a high prevalence of keloid development in certain ethnicities. Individuals from African, Hispanic, and Asian backgrounds have a higher likelihood of developing keloids when compared to Caucasians. Keloids are known to lack spontaneous regression and have a high rate of recurrence after removal, thereby causing a cosmetic problem that affects people physically and emotionally. Keloids commonly occur after burns, tattoos, piercings, and deep wounds; however, in rare cases, they may develop after minimally invasive procedures. This case describes the experience of a 48-year-old African American male who underwent a thyroid fine needle aspiration biopsy and subsequently developed a keloid in the neck region. This report aims to explore this unique occurrence, highlight the interplay between epidemiology, race, and genetics in influencing the development of keloids, and review the management strategies for neck keloids.

## Introduction

The term keloid was initially derived from the Greek word "cheloid" in the 1800s, which translates into "crab claw" [1]. Keloids appear as firm, pruritic, and rubbery nodules that form pedunculated lesions if their base is narrow or a more broad-based plaque if their base is wide [2]. In contrast to hypertrophic scars, they extend beyond the wound margins and invade the surrounding skin [1]. They have a propensity for locations such as the ear lobes, back of the head, upper chest and back, and flexural regions of the body [2]. Keloids are a complication of aberrant wound healing that greatly affects one’s quality of life. Symptoms include pain, itching, a sensation of tightness, irritability, and limitations in movement, depending on the region of the body involved [3].

Keloid formation is highly associated with ethnicity, with an estimated prevalence of 4.5% to 16% in persons with darker skin pigmentation [4]. Keloid development is more common in individuals of African, Hispanic, and Asian ethnic backgrounds and is associated with higher rates of keloid-related medical visits than in Caucasians [5]. While keloids may have a hereditary component, most cases are sporadic and do not show distinct inheritance patterns [6]. Although keloids have high recurrence rates and do not regress spontaneously, new treatment options are being developed. Keloid formation is a rare complication of a thyroid biopsy, and there is limited evidence in the medical literature reporting such cases. Keloids are more commonly associated with other types of skin trauma, such as tattoos, piercings, burns, and other surgical incisions [7]. 

Thyroid cytopathology is usually performed using fine needle aspiration cytology (FNAC), which is a minimally invasive procedure with low complication rates and faster recovery times [8]. Although individual healing times vary based on factors such as the size and location of the nodule, FNAC has mean healing times between one and two days [9]. While any procedure that causes skin trauma has the potential to result in keloid formation, the risk of keloid development is generally considered to be especially low for thyroid biopsies [8]. We present the case of a 48-year-old African American male with a history of multiple piercings and tattoos who did not develop any keloids after these procedures; however, after undergoing FNAC of a thyroid nodule found incidentally, he developed a neck keloid.

## Case presentation

We present the case of a 48-year-old African American male who presented to the outpatient clinic for a keloid evaluation. The patient’s past medical history was insignificant apart from hypertension. He stated that two years prior, during his annual physical examination, a mass in the midline neck area was discovered. After evaluation, it was discovered that the mass was in his thyroid, and an FNAC was taken. Results were negative for malignancy, per the patient's recollection. Three months after the test, the patient noticed the development of a keloid at the site of the biopsy, which continued to grow over the next two years. Upon further questioning, the patient stated that he had a history of having tattoos and piercings with no previous occurrence of keloid formation. He also denied having a positive family history of keloid formation.

**Figure 1:**
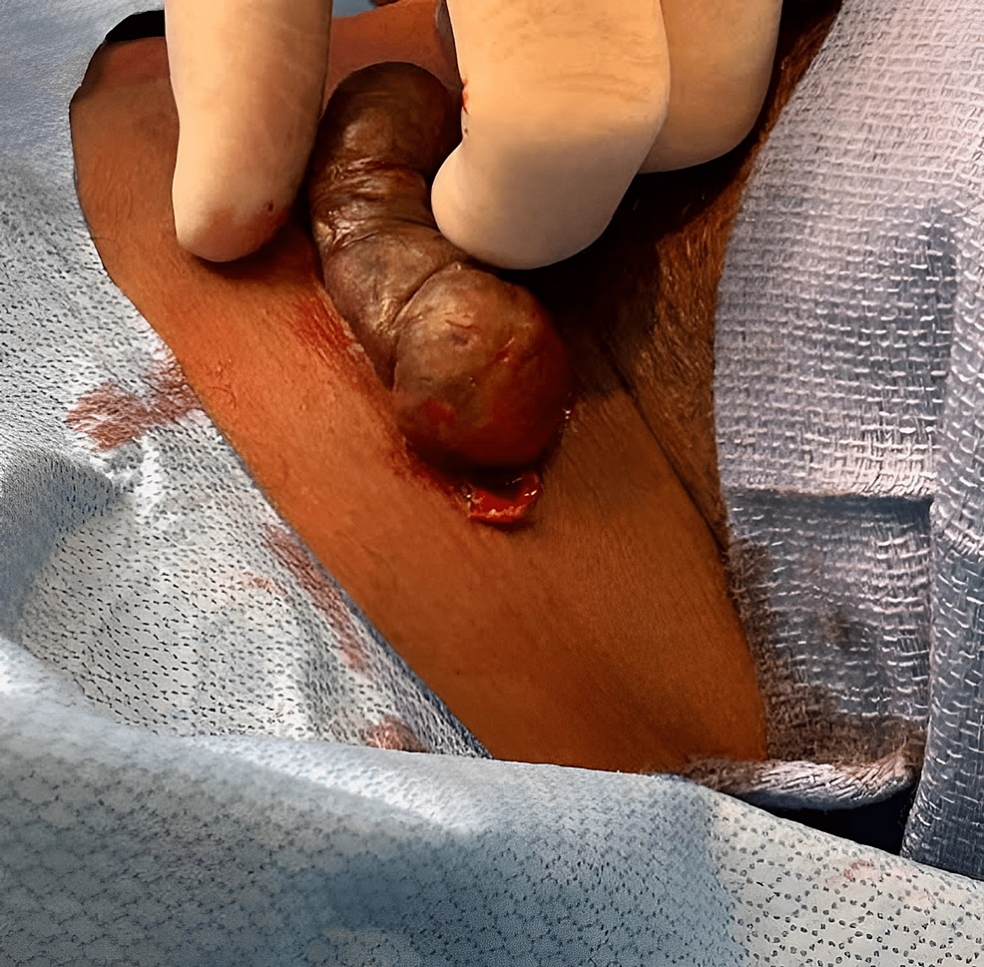
Large keloid located in the midline neck region after thyroid fine needle aspiration

Upon physical examination, a shiny, firm, hyperpigmented lesion was visualized in the midline area of the neck, measuring 10 x 4 cm. Figure 1 below shows the keloid that formed from the thyroid biopsy.

The patient underwent keloid removal surgery. The keloid was excised in an elliptical fashion at the level of the skin, the wound was closed in layers, and Triamcinolone Acetonide was injected subcutaneously into the wound to decrease inflammation and pain. The surgery was well tolerated with no complications, and after an unremarkable postoperative period, the patient was discharged from the hospital on the same day. 

## Discussion

Keloid formation is suggested to be due to an exaggerated and prolonged inflammatory response in response to skin injury [10]. This leads to sustained expression of various inflammatory mediators and growth factors such as Transforming Growth Factor Beta-1 (TGF- β1), Vascular Endothelial Growth Factor (VEGF), and Platelet-Derived Growth Factor (PDGF) [9]. Subsequently, increased fibroblast proliferation and activity and collagen formation and deposition in the extracellular matrix (ECM) occur [3]. Studies show that collagen is produced at a rate that is 20 times greater in keloid formation than in healthy skin and three times higher than in hypertrophic scars [5]. 

There is an association between melanin and keloid development. This is supported by the following: (i) the incidence of keloid formation is five to 15 times higher in black people than in other races, (ii) persons with albinism rarely develop keloids, and (iii) the incidence of keloid formation is lower on the palms and soles, where there are fewer melanocytes [11]. After the skin is injured, cytokine release leads to melanocyte proliferation and melanin production in the area [11]. Due to epidermal layer damage, fibroblasts grow into the injured tissue, contact the melanocytes, and begin proliferation. Therefore, it is assumed that the more melanocytes in the area, the higher the fibroblast proliferation, resulting in keloid formation [12]. Most cases of keloids are sporadic; however, some familial cases indicate a genetic influence. A study by Marneros et al. explored the genetic susceptibility for keloid formation in two families with keloids developing in an autosomal dominant pattern [13]. They found keloid susceptibility linked to chromosomes two and seven [13]. In this case, the patient had a negative family history of keloids, indicating a sporadic nature of development.

Environmental factors such as mechanical tension or stress and injury to the skin are the main contributors to keloid formation and progression [14]. Keloids may develop after burns, body piercings, especially ear piercings, acne, chicken pox, tattoos, and deep wounds like puncture wounds [14]. Some studies show that inflammatory skin conditions such as acne vulgaris, varicella infection, and bacterial folliculitis can trigger keloid formation [14]. In this case, the patient had a history of multiple tattoos along the arms and chest and ear piercings with no incidence of keloid formation. Clinically, keloids present as slow-growing, benign, dense, raised fibrous tissue that extends beyond the original wound or inflammatory response margin [6]. They are commonly seen around the age of 10 to 30 years old and can be shiny, hairless, skin-colored, hypo- or hyper-pigmented, or erythematous [6]. Keloids can develop anywhere on the skin; however, the most commonly affected locations are the ear, chest, chin, neck, deltoid, upper back, shoulders, and lower legs. In this case, the patient’s keloids were shiny, firm, and hyperpigmented, and they developed in the neck region.

Every year, more than 700,000 biopsies are performed in the United States [15]. This minimally invasive diagnostic test allows the analysis of tissue cells from organs or tumors [15]. There are different types of biopsy procedures depending on the area of interest. FNACs evaluate swellings or lumps just under the skin, like thyroid nodules or suspicious lymph nodes. Pain, bleeding, and bruising are common complications of FNACs of the thyroid [16]. Others include swelling of the thyroid or neck region, short-term hoarseness due to temporary nerve injury, difficulty swallowing, and transiently increased thyroid function [16]. Scarring and keloid formation are rare occurrences after biopsy, with an increased risk when the biopsy is performed on the neck, back, or chest area [11]. In this case, a thyroid nodule was felt, and the patient underwent an FNAC without any significant complications; however, months later, a large keloid formed. Large keloids, despite their predisposition for the neck area, are fairly uncommon and usually develop after a higher degree of injury than an FNAC. There is a paucity of literature exploring the risk factors and natural history of large neck keloids. A study by Tirgan et al. evaluated 82 patients with neck keloids and discovered that 65 patients-all African Americans-had keloidal lesions elsewhere on the body [17]. They suggested that large neck keloids may be race-specific since they are almost exclusively seen in African Americans [17]. 

The diagnosis of keloids is clinical and is based on the patient’s history and physical findings; however, a biopsy may be conducted to rule out other differentials such as hypertrophic scar, acne keloidalis, keloidal dermatofibroma, or skin cancer [6]. Increased whorls of thickened, randomly organized Type I and Type III collagen fibers and hyalinized keloidal collagen bundles are characteristic features found on biopsy [4]. They are seen in up to 55% of lesions [4]. These differ from hypertrophic scars, which don’t extend beyond the wound margin and have an organized pattern of Type III collagen on biopsy [9]. Studies suggest that the most effective management of keloids involves combination therapy with two or more treatment modalities [18]. Some treatments include (i) tension-free wound care with the use of silicone sheeting in cases of accidental skin trauma or as a part of postoperative wound care, (ii) intralesional steroid use with Triamcinolone Acetonide, which is the first-line treatment and associated with a response rate between 50% and 100%, (iii) cryotherapy, which is inexpensive but associated with post-treatment pigment alterations and (iv) surgical excision, which immediately relieves the emotional burden; however, it is associated with a high recurrence rate of 45%-100% [4]. An article by Thornton et al. suggests that intralesional steroid use plus intralesional cryotherapy has the most promising results for nonauricular keloids, while surgical excision plus compression therapy should be considered first-line for auricular keloids [18]. Other successful treatment options include topical imiquimod following surgical excision, intralesional bleomycin, intralesional 5-fluorouracil, and pulsed dye laser [14]. 

This patient had no family history or prior occurrence of keloid formation despite having multiple tattoos and piercings; however, a large keloid developed after a minimally invasive diagnostic test. This case is a unique occurrence, and physicians should be aware that even FNACs may lead to large neck keloid formation, especially in African American patients. 

## Conclusions

Keloids are a problematic cosmetic result of skin trauma; however, this case showcases a rare occurrence. Persons of African American ethnicity and those with positive family histories are at a higher risk for keloid formation. They should be aware of this risk and exercise caution even when undergoing minimally invasive procedures such as biopsies. Due to the sporadic nature of keloid formation, persons with previous tattoos and piercings can develop keloids at any time. Physicians should be aware of the fact that keloids can arise after mild trauma and should inform high-risk patients of this possibility before performing diagnostic tests that require puncturing the skin. Various treatment strategies can be utilized in the management of keloids. Treatment should be tailored uniquely to each patient and be based on lesion size, safety, risk of recurrence, and the patient’s goal for treatment to improve their quality of life. 
